# Acidic Pretreatment of Wheat Straw in Decanol for the Production of Surfactant, Lignin and Glucose

**DOI:** 10.3390/ijms13010348

**Published:** 2011-12-28

**Authors:** Sinisa Marinkovic, Jean Le Bras, Véronique Nardello-Rataj, Mickaël Agach, Boris Estrine

**Affiliations:** 1 Agro-Industrie Recherches et Développement “Green Chemistry Department”, F-51110 POMACLE, France; E-Mails: s.marinkovic@a-r-d.fr (S.M.); m.agach@a-r-d.fr (M.A.); 2 Institut de Chimie Moléculaire de Reims, UMR 6229, CNRS-Université de Reims Champagne-Ardenne, UFR des Sciences exactes et naturelles, BP 1039, 51687 REIMS Cedex 2, France; E-Mail: j.lebras@univ-reims.fr; 3 Université Lille1, EA 4478 “Chimie Moléculaire et Formulation”, F-59655 Villeneuve d’Ascq Cedex, France; E-Mail: veronique.rataj@univ-lille1.fr

**Keywords:** wheat straw, biomass, glycosylation, surfactants, lignin, decyl pentosides

## Abstract

Wheat straw is an abundant residue of agriculture which is increasingly being considered as feedstock for the production of fuels, energy and chemicals. The acidic decanol-based pre-treatment of wheat straw has been investigated in this work. Wheat straw hemicellulose has been efficiently converted during a single step operation into decyl pentoside surfactants and the remaining material has been preserved keeping all its promises as potential feedstock for fuels or value added platform chemicals such as hydroxymethylfurfural (HMF). The enzymatic digestibility of the cellulose contained in the straw residue has been evaluated and the lignin prepared from the material characterized. Wheat-based surfactants thus obtained have exhibited superior surface properties compared to fossil-based polyethoxylates decyl alcohol or alkyl oligoglucosides, some of which are largely used surfactants. In view of the growing importance of renewable resource-based molecules in the chemical industry, this approach may open a new avenue for the conversion of wheat straw into various chemicals.

## 1. Introduction

**Scheme 1:**
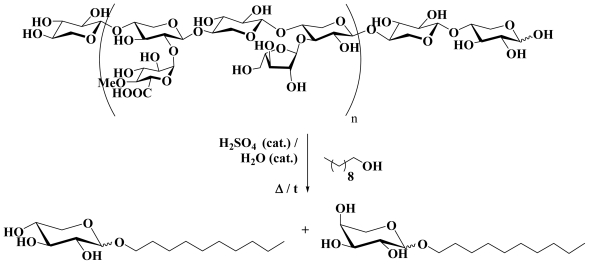
Wheat straw hemicellulose conversion into decyl glycosides (glucosides and furanosides are omitted for clarity).

Hemicellulose, the second most abundant source of biomass next to cellulose, is currently regarded as a promising alternative to fossil chemicals. Since it cannot be digested by human beings, its use, unlike corn and starch, would not induce any negative impact on food supplies [1]. Wheat straw represents a valuable source of hemicelluloses, cellulose and lignin; it is an available co-product of wheat crop that has strong potential tonnages. In the European Union, 1 to 5 million tons could be collected per year [2]. In a context where interest is constantly growing for integrated lignocellulosic biorefineries, there is a need for a better use of the crop-sourced hemicellulose and for an improvement of the added value of such concepts [3]. For example, when lignocellulosic ethanol is considered, hemicelluloses are potentially a bottleneck as the industrial fermentation of pentoses has not yet been achieved in a cost-efficient way [4]. The chemical industry is also looking for new feedstock for their production and pentoses could play a role on this field [4]. As an example, the surfactant industry started proposing some green surfactant solutions in the last decades. However, most of them are based on food related sugars such as glucose from starch and remain poorly used due to their price. This is the case for alkylpolyglucosides (APG). Alternatively, pentose-based surfactants produced from agricultural wastes are gaining interest in the surfactant market for detergent and cosmetic applications as they display high detergent properties and a better ecological profile [5]. The work presented in this paper describes a new pretreatment process of wheat straw for producing mixtures of n-decyl-pentosides with good surface activities. This process directly converts wheat straw hemicelluloses in acidic decanol phase (Scheme 1). This process differs from usual biomass fractionation methodologies that are inherent to prepare sugars in pure forms for chemists. The last process consists of several operation units. The first step is the polysaccharides hydrolysis, followed by a second step of sugar monomers purification. Finally, sugars are chemically transformed [6]. The method developed herein contributes to broaden the use of pentose-based surfactants in the detergent industry. It should allow a substantial cost saving as glycosylation of pentoses occurs during pretreatment of biomass. Furthermore, the wheat straw residue is also preserved and can be submitted to an enzymatic hydrolysis of cellulose for both production of glucose and lignin.

## 2. Results and Discussion

### 2.1. Pretreatment

The pretreatment study has been realized with the aim to valorize to highest part of hemicelluloses into alkyl pentosides. Firstly, the reaction was carried out at 90 °C and under atmospheric pressure in the presence of 14 weight equivalents of *n*-decyl alcohol, 0.7 wt % of H_2_SO_4_ as catalyst, and 0.7 wt % of H_2_O as co-catalyst (Table 1, Entries 1–6). These mild conditions can be compared with the high temperatures and pressures (165 °C, 20 bars) commonly used for the starch transglycosylation [7]. Dilute acid processes developed for the fractionation of biomass usually involve high amount of water or organic solvents. Very few processes in which the biomass loading is up to 15 wt % have been described. The main reason is the difficulty to maintain good stirring conditions. In these new experimental conditions, the biomass loading was limited for the same reason. Here, the *n*-decyl-pentosides formation slowly occurs (Table 1, Entries 1–6). After 30 min (Table 1, Entry 1), arabinose contained in hemicelluoses is glycosylated. This is explained by the good availability of the arabinofuranose moieties along the hemicellulose chains. The fast and quantitative formation of arabinosides is in accordance with the literature results [8]. On the other hand, the *n*-decyl-xylosides concentration continuously increases until 4 h of reaction (Table 1, Entries 1–5). The production of a small quantity of *n*-decyl glucosides has also been noticed. Concerning the final weight ratio between xylosides and arabinosides (4.2), it is possible to compare it with the ratio between xylose and arabinose contained in the starting material (4.8 see Table 2). The presence of a very low concentration of sugar monomers (xylose mainly), and *n*-decyl-pentosides in their α and ® anomeric forms have been noticed. It is also important to mention that without added water as a co-catalyst, the reaction does not proceed efficiently (less than 25% of pentosides after 2 h of reaction). The fact that the absence of added water limits the yields of glycosides would mean that a hydrolysis reaction is a limiting step in a favorable “hydrolysis—glycosylation” mechanism [8]. Experiments were also carried out at higher temperature (Table 1, Entries 7–12). A substantial improvement has been observed since 95.2% of *n*-decyl-pentosides yield could be reached within 2 h. Afterwards, the yield did not evolve significantly, proving the good stability of glycosides under these experimental conditions. In these experiments, the *n*-decyl-glucosides concentration was also raised to 14.3% corresponding to a wheat straw cellulose transformation of about 10%. When temperature was fixed at 125 °C (Table 1, Entries 13–15), the yield of decyl pentosides was no longer improved due to a degradation of the surfactants. The productivity of the process could be improved by carrying out the reaction in only 7 weight equivalents of alcohol. At 90 °C (Table 1, Entries 15–17), the reaction actually proceeds faster than experiments performed with 14 equivalents of alcohols. A yield of 55.6% is achieved in 3 h (Table 1, Entry 17), whereas in diluted conditions, 5 h are required to obtain a similar result (Table 1, Entry 6). At 109 °C (Table 1, Entries 18–21), 92.7% of pentosides are formed, but a degradation of glycosides appears after 2 h (Table 1, Entries 20 and 21). This degradation can be explained by the high loading of biomass and thus, the higher amount of acid catalyst favouring hydrolysis reactions.

### 2.2. Residue Analysis and Glucose and Lignin Production

The chemical composition of the straw residue collected in the best pre-treatment trial (conditions of the Entry 9, Table 1) was analyzed and compared to the starting wheat straw [9]. Results are presented in Table 2, and show that the residue recovered is still rich in cellulose. The low amount of residual xylan and the absence of araban in the residue confirm the good reactivity of hemicellulose. The residue was then submitted to enzymatic hydrolysis [10]. Cellulose digestibility of the residue was 53% (production of glucose) after 72 h, based on the initial cellulose content. This value is typically in the range of cellulose digestibility of a dilute acid pre-treated wheat straw (47% [11]) and obviously higher than a non pre-treated wheat straw (17% following same procedure [10]). This is interesting because, in our method, the glucose filtrates obtained by enzymatic digestion are free of any pentoses streams that are known to be more difficult to convert into ethanol through fermentation processes.

Lignin prepared from the same batch of straw residue was analyzed [12]. The molecular weight was measured by gel permeation chromatography [13] and compared to a reference organosolv lignin obtained from wheat straw by solubilisation in organic acid mixture (acetic and formic acid) and recovered by a precipitation procedure [14]. The lignin from the surfactant process is obviously different as it is an organic insoluble material recovered from the pre-treated wheat straw residue. This difference is clearly displayed by weight-average molecular mass (Mw) of the lignin produced from the surfactant process that is higher than the Mw of the organosolv lignin (Table 3). The research works found in literature support the utilization of high Mw lignin for the synthesis of modified phenolic resins [13]. The high Mw is potentially explained by the presence of impurities (cellulose) or by a structure difference (more guaiacyl units). This is supported by the FT-IR spectra of the two lignin samples (see supplementary information) that display some band differences in the 700–1200 cm^−1^ region.

### 2.3. Surfactant Properties

We isolated decyl glycosides from the filtrate (same conditions described in trial 9 of Table 1) after *n*-decyl alcohol excess had been distilled off. 58 g of a crude *n*-decyl-glycoside mixture were thus recovered (see supplementary information) containing a residual alcohol content of 0.3%. Pollutants such as sulphated ashes and proteins were analyzed (respectively 2.1 wt %, and 0.8 wt % in the crude mixture of glycosides; Proteins content obtained following Kjeldhal method).

The surface activity (*i.e.*, critical aggregation concentration CAC and surface tension at the CAC, ©_CAC_) of the glycoside mixture thus obtained (see detailed composition in supplementary information) was then studied and compared to other well-known alkyl glycosides and a broadly used polyethoxylated alcohol (Table 4). In order to assess the usefulness of the glycoside mixture in detergency, foaming properties and wetting power were also evaluated following Ross Miles [15] and Draves tests [16].

The performance properties of surfactants, in particular interfacial properties and behaviour in solutions (for example, phase behaviour), are essentially attributable to specific physicochemical effects [20]. The surface tension of alkyl glycosides from wheat straw was investigated and compared to alkyl glycosides obtained by Fisher’s glycosylation [21] and with octaethylene glycol mono *n*-decyl ether [19]. From all surfactants studied, the critical aggregation concentration values (CAC) of pentoside containing compositions are the lowest (301 and 483 mg·L^−1^). In other words, the pentoside compositions exhibit the best efficiencies. This behaviour is explained by the fact that *n*-decyl-pentosides are more hydrophobic substances and thus tend to aggregate at a lower concentration [22,23]. Obviously, due to the presence of impurities in the glycosides directly obtained from biomass, the CAC is higher than the one of pure d-xylosides. It is clear that inorganic pollutants, proteins and probably soluble lignin lower the concentration of surface active glycosides in the crude composition. Although electrolytes are known to induce salting out effects, the surface activity of the impure glycoside composition remains slightly lower than the pure decyl xylosides [24]. However, the efficiency of the biomass derived glycosides is equivalent to the ethoxylated surfactant (483 mg·L^−1^ for pentosides and 511 mg·L^−1^ for octaethylene glycol mono decyl ether). All glycoside compositions display similar effectiveness (surface tensions comprised between 26 and 29 mN·m^−1^), superior to the one of the decyl polyethoxylated alcohol which lowers surface tension till 37 mN·m^−1^. Foam power and stability of the *n*-decyl-glycosides from wheat straw are not dramatically affected by impurities (similar to d-xylosides). However, the wetting power is slightly lower than pure xylosides but remains acceptable compared to commercial oligoglycosides. So, it is possible to envisage the use of the surfactants obtained following the process described in this paper, and this without further purifications.

## 3. Experimental Section

### 3.1. Raw Material

The wheat straw was supplied by local farmers in Champagne Ardennes region (FRANCE). The dry matter content of the wheat straw was 92 wt %. The carbohydrate content was determined according to NREL (National Renewable Energy Laboratory) analytical methods [9]. The compositions are given in Table 2.

### 3.2. Pretreatment of Wheat Straw

In a typical pretreatment procedure, 700 g of decyl alcohol containing 0.7 wt % of sulfuric acid, 0.7 wt % of deionized water, and 50 g of chopped straw are loaded in a reaction vessel. The medium is warmed up to the reaction temperature. When 100 g of straw are loaded, sulfuric acid and water concentration in alcohol are 1.4 wt %. The remaining straw is filtered off on a paper filter and the crude filtrate analyzed after neutralization with 8 g of a 30.5 wt % NaOH solution. The residual material is washed with acetone, dried at 90 °C under vacuum and weighed. As the reaction products are rarely commercially available, alkyl glycoside standards were prepared by Fisher’s glycosylation of d-xylose, and l-arabinose in *n*-decyl alcohol excess. Each was purified using column chromatography. Their purity was checked by ^13^C NMR, ^1^H NMR and GC-MS [8]. The GC calibration was performed using these purified products with an accuracy of 97% minimum (see Supplementary Information).

### 3.3. Production of Glucose by Enzymatic Digestibility of Cellulose

Enzymatic hydrolysis of cellulose contained in wheat straw residues into glucose has been done following litterature protocole [10]. 16 g of wheat straw or pretreated wheat straw recovered from trial of Entry 9, Table 1, is loaded in a reaction vessel containing enzymes (total volume of 800 mL). Each reaction vessel was sterilized at 121 °C for 20 min prior to the addition of the enzymes and straw or straw residue. The enzymes were added at the following amounts: 3.7 g Celluclast 1.5 L (60 FPU/g, 30 ®-glucosidase IU/g) (Novozymes A/S), 0.8 g Novozyme 188 (500 β-glucosidase IU/g) (Novozymes A/S) and 0.8 g Multifect Xylanase (43 g protein/mL) (Genencor International Inc.). Enzymatic hydrolysis was performed at 40 °C for 72 h. The sample is immediately filtered through a 0.2 μm sterile filter and frozen to prevent further hydrolysis. Duplicate batches were run to verify the results.

### 3.4. Lignin Preparation and Caracterisation

Lignin was prepared from residues by chemical hydrolysis with concentrated sulphuric acid following literature procedure [12]. The molecular weight was measured by gel permeation chromatography and FT-IR spectra obtained following literature method [13]. The residue samples obtained following the pretreatment method were subjected to acetylation in order to enhance their solubility in organic solvents used in GPC. The residues were placed in an acetyl anhydride/acetyl acid mixture (1:1 by weight) containing sodium acetate as a catalyst (0.5 equivalents per mole of acetyl anhydride), with a final residue concentration of 20 wt %. Reaction was carried out at room temperature for 48 h and then refluxed for 1 h. Products obtained by precipitation in ice-cold water with 1% HCl were filtered, washed with distilled water and dried in the same way as the residue samples. The gel permeation chromatography experiments were performed at 20 °C with a set up consisting of an ASI-100 Automated Sample Injector (Dionex), a P680 HLPC pump (Dionex), two PLgel MIXED-E columns (Varian, dimension: 300 × 7.5 mm, internal diameter: 3 μm), a refractometer (RI-101, Shodex) and a thermostat (Thermostated column compartment TCC-100). The mobile phase was made of THF (Chromasolv®, Sigma Aldrich) and the flow rate was 1 ml.min-1. The weight distribution comparison was established with (PolyEthylene Glycol, PEG) standards (Varian).

### 3.5. Physicochemical Properties

Measurement of surface tension, foaming and wetting ability were performed following the same protocols as described in literature [8,22,23].

## 4. Conclusions

In summary, we have reported a new pretreatment methodology for the direct and selective conversion of wheat straw hemicelluloses into glycoside surfactants. The remaining straw material is preserved keeping all its promises as potential feedstock for fuels or value added platform chemicals like HMF. The wheat-based surfactants thus obtained without further purification have shown superior surface properties compared to fossil-based decyl polyethoxylated alcohol or alkyl oligoglucosides, some of which being largely used as surfactants. In view of the growing importance of renewable resource-based molecules in detergents and cosmetics industries, this approach may open a new avenue for the production of green surfactants.

## Supplementary Information



## Figures and Tables

**Table 1:** Pretreatment of wheat straw in sulphuric acid decanol phase.

Entry	Temp. (°C)	Wheat straw loaded (g)	Time(h)	Yield pentosides	Ara	Xyl	Glu

				(%) a	(%) b		
1	90	50	0.5	13.2	40	60	0
2	90	50	1	23.8	33.3	66.7	0
3	90	50	2	34.4	23.1	76.9	0
4	90	50	3	42.3	17.7	76.5	5.9
5	90	50	4	47.6	15.8	79	5.3
6	90	50	5	55.6	18.2	77.3	4.6
7	109	50	0.5	68.8	19.4	67.7	16.1
8	109	50	1	87.3	21.6	67.6	10.8
9	109	50	2	95.2	19.5	68.3	12.2
10	109	50	3	95.2	16.7	69.1	14.2
11	109	50	4	95.2	19.1	66.7	14.2
12	109	50	5	95.2	19.1	66.7	14.2
13	125	50	0.5	74.1	16.1	74.2	9.7
14	125	50	1	74.1	15.6	71.9	12.5
15	125	50	2	63.5	14.3	71.4	14.3
16	90	100	1	41.4	16.7	80	3.3
17	90	100	2	45.7	14.7	79.4	5.9
18	90	100	3	55.6	12.2	82.9	4.9
19	109	100	0.5	65.6	17.7	72.6	4.9
20	109	100	1	75.6	15.3	74.6	10.2
21	109	100	2	92.7	15.3	75	9.7
22	109	100	3	77	14.8	73.8	11.5

a Yield of monopentosides (glucosides not considered) are determined by GC method [8];

b Arabinosides, Xylosides and Glucosides: GC distribution in the crude filtrate (see Supplementary Information).

**Table 2:** Composition of wheat straw and residue a.

	Araban (Wt %)	Xylan (Wt %)	Glucan (Wt %)
Starting wheat straw	5	24	39
Recovered residue	0	0.7	52

a % weight of sugars determined by CLHP using internal standard (see supporting information). Analysis done on the filtrate obtained after applying known procedure for hydrolysis of wheat straw [9].

**Table 3:** Molecular weight of lignin [13].

	Mn	Mw	IP = Mw/Mn
Reference lignin (g/mol)	909	2001	2.2
Surfactant process lignin (g/mol)	905	3433	3.8

**Table 4:** Surface properties, foaming and wetting power of glycoside compositions from wheat straw, d-xylose and d-glucose.

Surfactant composition	CAC (mg·L^−1^)	γ_CAC_ (mN·m^−1^)	Foam volume at t = 0 (mL) (Stability at 20 min (%))	Wetting time (s)
wheat straw decyl–glycosides *	483	28	480 (78)	45
decyl xylosides from d-xylose	301	28	480 (75)	23
decyl b-d-glucopyranoside [17]	994	29	-	-
octyl/decyl polyglucosides [18]	963	26	450 (75)	196
octaethylene glycol mono decyl ether [19]	511	37	-	-

* Table 1, Entry 12.
