# Supplementary material for: Acidic Pretreatment of Wheat Straw in Decanol for the Production of Surfactant, Lignin and Glucose
Source: Int J Mol Sci. 2011 Dec 28;13(1):348–57. doi: 10.3390/ijms13010348 (PMC3269690; doi:10.3390/ijms13010348)
Supplement: Supplementary file 1 [file ijms-13-00348-s001.pdf]

Supplementary Information

1. NMR, FT-IR and MS/HRMS

NMR spectra were recorded with a Bruker AC 250 (250 MHz for ^{1}H and 62 MHz for ^{13}C). Chemical shifts are given in ppm relative to the residual solvent signal (pyridine-d_5).

IR spectra were recorded on a Nicolet AVATAR 320 FT-IR.

MS and HRMS were obtained on a hybrid tandem quadrupole/time-of-flight (Q-TOF) instrument, equipped with a pneumatically assisted electrospray (Z-spray) ion source (Micromass, Manchester, UK) operated in positive mode (EV = 30 V, 80 ℃, flow of injection 5 mL/min).

All decyl glycosides used as standards are known products synthesised and characterised as previously described [1].

2. Materials for GC Chromatography

docosanol, anhydrous pyridine (ACROS, 99 +%), 1,1,1,3,3,3-hexamethyldisilazane (HMDS) (ACROS, 98%), Chlorotrimethylsilane (TMSCl) (Aldrich, 98%), tert-butylmethylether (TBME), (ACROS, 99%)

3. Preparation of the Sample

A 50 mL flask was charged with 1000 mg (precise amount) of the glycosides solution and 100 mg (precise amount) of docosanol. 10 mL of anhydrous pyridine was then added and the mixture was stirred until complete dissolution (~12 h) .5 mL of HMDS and 3 mL of TMSCl were then added. The mixture was stirred for 10 min, then the solvent was evaporated under vacuum (1 mbar) at 55 ℃. The crude residue was then solubilized in 6 mL of TBME and filtered.

4. Gas Chromatography

* Apparatus: Shimadzu GC-14B/* Column: Track 2 CP-sil 13 CB (Varian) Inside diameter: 0.32 mm; Thickness: 1.2 m; Length: 25 m/* Chromatographic conditions: File 7, Range 0; Oven: 230 ℃: 32 min; 20 ℃/min, 300 ℃; 300 ℃: 5 min; - Injector: 250 ℃ - Sensors (F.I.D.): 340 ℃;

* Injection: 0.4 µL;

* Carrier gas: Helium 100 kPa;

STANDARD CALIBRATION with docosanol as an internal reference:

**Standard chemical**	**Calibration curve y = aX +b**		**R^2 Regression coefficient**
	**a**	**b**	
C10 arabinosides	1.2951	−0.0021	0.9947
C10 xylosides	1.8945	−0.0475	0.9958
C10 glucosides	1.0215	−0.0183	0.9973

5. Separation of Sugars: Liquid Chromatography on Ion Exchange Column in Hydrogen Form

Column: Aminex HPX 87H 300 × 7.8 mm (Supplier: Biorad, ref 125-0140);

Pre-column: Aminex HPX 87H 30 × 4.6 (Supplier: Biorad, ref 125-0129);

Column temperature: 45 ℃;
Eluent: 0.01 N H2SO4 (1.4 mL concentrated H_2SO_4 for 5 L);
Flow rate: 0.6 mL/min (peak pressure < 120 bar);
Injection Volume: 20 μL;
Detection: Refractometer;
Calibration: standard range of 0.1 to 1 g/L sugar;
Example of separation conducted:

Sugars	RT (min)
Glucose	9.36
Xylose	10.02
Mannose	9.94
Galactose	9.98
arabinose	10.94

6. Separation of Glycosides

The crude glycosides mixture (58 g) was obtained after *n*-decyl alcohol was distilled off at 160 ℃ and 1 mbar. This quantity of glycosides was recovered from two trials performed following conditions described at Entry 9, Table 1.

	Weight	GC purity	GC Glycosides distribution (Wt. %)		
			Arabinosides	Xylosides	Glucosides
Starting wheat straw (wet material)	100 g				
Crude Glycosides Recovered after decanol distillation	58 g	95%	18	63	19

FT-IR: Reference Lignin.

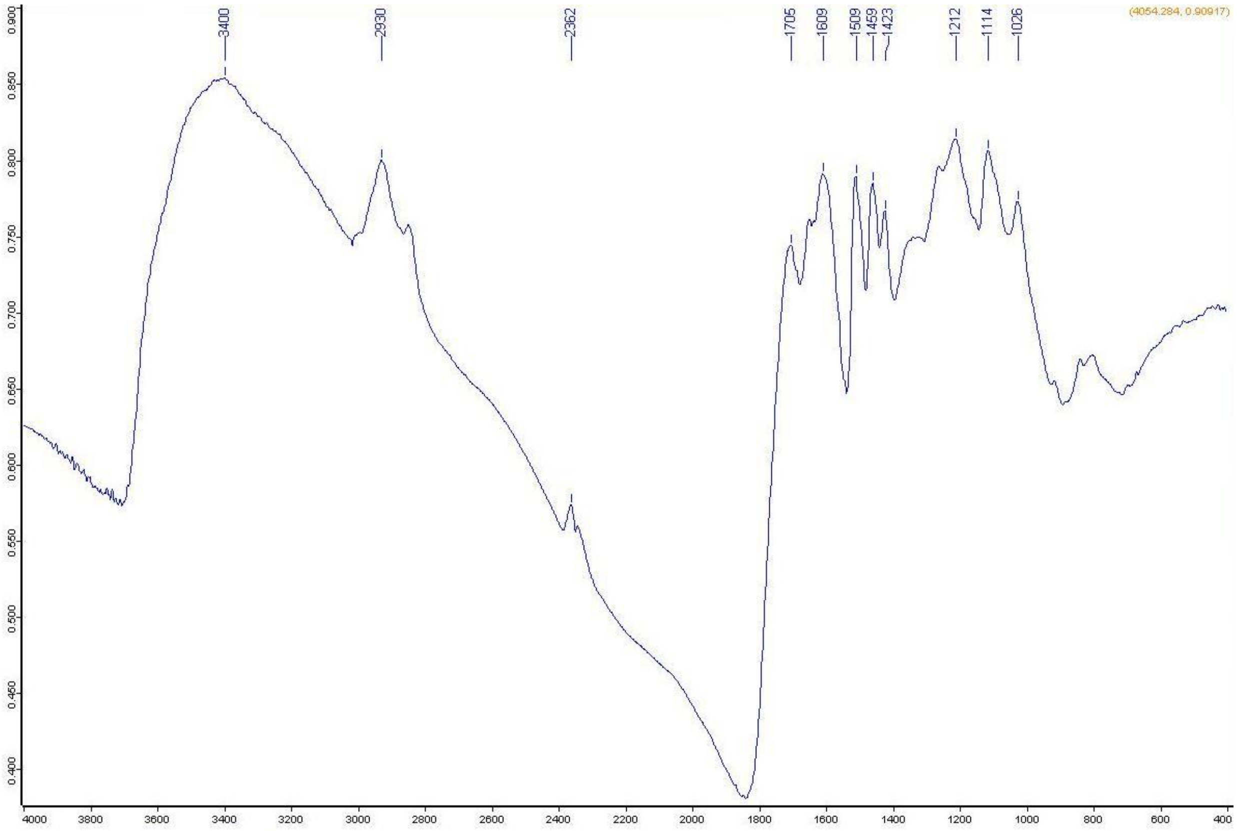

FT-IR Lignin from surfactant process.

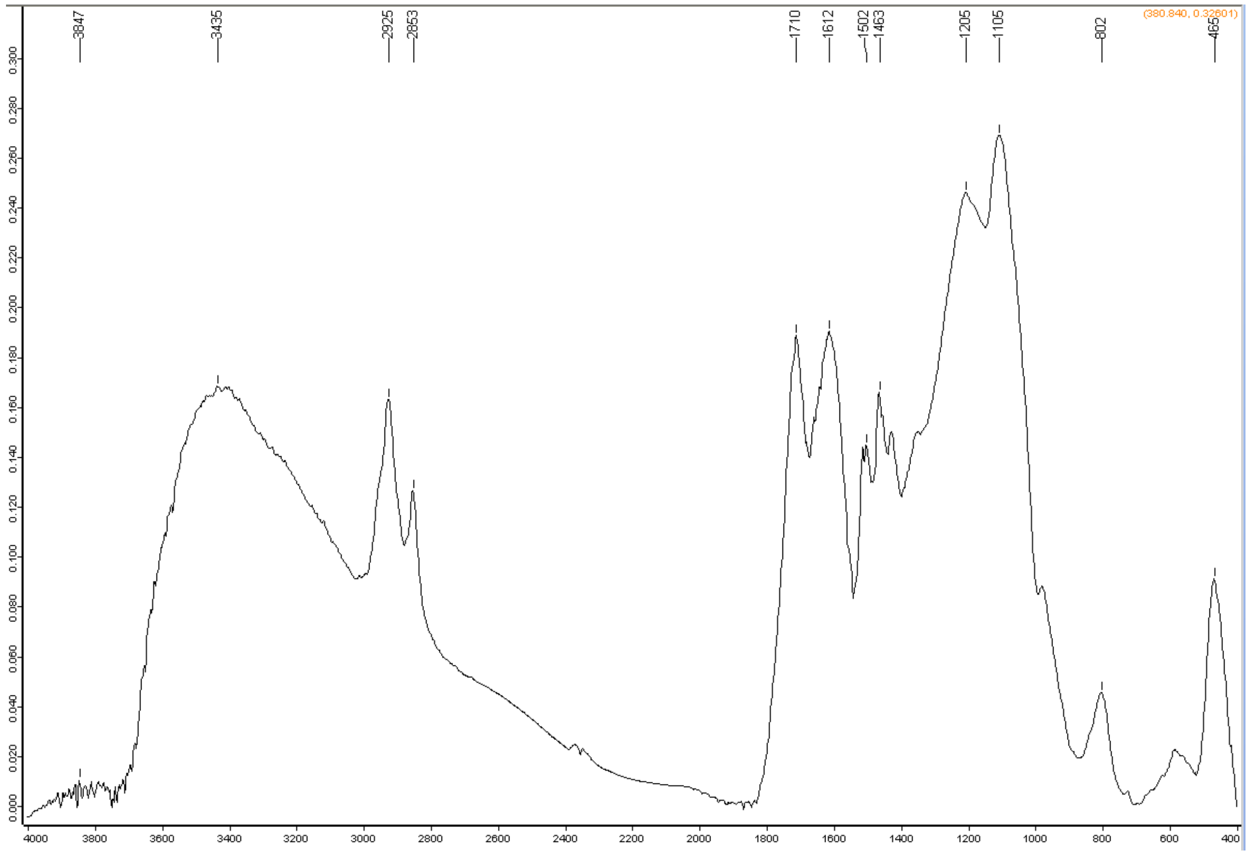

Reference

1. Bouxin, F.; Marinkovic, S.; le Bras, J.; Estrine, B. Direct conversion of xylan into alkyl pentosides. *Carbohydr. Res.* **2010**, *17*, 2469–2473.
